# Simultaneous Recovery of Matrix and Fiber in Carbon Reinforced Composites through a Diels–Alder Solvolysis Process

**DOI:** 10.3390/polym11061007

**Published:** 2019-06-06

**Authors:** Giovanni Fortunato, Luca Anghileri, Gianmarco Griffini, Stefano Turri

**Affiliations:** Department of Chemistry, Materials and Chemical Engineering “Giulio Natta”, Politecnico di Milano, Piazza Leonardo da Vinci 32, 20133 Milano, Italy; giovanni.fortunato@polimi.it (G.F.); luca8anghileri@gmail.com (L.A.)

**Keywords:** Diels–Alder, solvolysis, fiber recovery, carbon fiber, composite, epoxy, self-healing

## Abstract

Efficient and comprehensive recycling of fiber-reinforced thermosets is particularly challenging, since the irreversible degradation of the matrix component is necessary in order to separate the fiber component in high purity. In this work, a new approach to fully recyclable thermoset composites is presented, based on the thermal reversibility of an epoxy-based polymer network, crosslinked through Diels–Alder (DA) chemistry. Carbon fiber composites, fabricated by compression molding, were efficiently recycled through a simple solvolysis procedure in common solvents, under mild conditions, with no catalysts. Specifically, the purity of reclaimed fibers, assessed by thermogravimetric analysis and scanning electron microscopy, was very high (>95%) and allowed successful reprocessing into second generation composites. Moreover, the dissolved matrix residues were directly employed to prepare smart, thermally healable coatings. Overall, DA chemistry has been shown to provide a convenient strategy towards circular economy of thermoset composites.

## 1. Introduction

The increasing global demand for lightweight materials is generating a continuous growth of the composite industry. In particular, carbon reinforced composites are progressively entering novel application sectors such as the automotive field, in addition to more established markets like aerospace and wind power. Therefore, beside the technological challenge of ensuring greater volumes at competitive costs, the composite industry has to address the recycling issue [1], as a dramatic increase in waste from end-of-life composites is foreseen in the next few years, landfilling being progressively forbidden [2]. 

Available technologies for carbon composite recycling are mainly focused on the recovery of the fiber component, while the resin component is generally considered waste due to its lower market value [3]. Composite recycling methods are generally grouped into three categories: Mechanical, thermal, and chemical processes. Mechanical recycling is probably the least expensive treatment, but at the same time it produces low quality recyclates. Since fibers are broken and not liberated, mechanical shredding and milling do not achieve a clean separation between fiber and resin. Thus, the obtained powders can only be proposed as cheap, low-performing fillers for new composites [4]. As a result, this method was not scaled to industrial level, at least for carbon-based composites. On the contrary, thermal treatments like pyrolysis were commercially exploited, since they allow to achieve high fiber purity [3]. In these approaches, the grinded or cut composite is treated at high temperature (typically 400–700 °C) in an inert or oxidative environment in order to selectively degrade the resin component into volatile products and char, which is then deposited on the fibers. The char residues can be minimized in oxidative or more severe conditions but at the expense of unavoidable worsening of mechanical strength. Comparatively, chemical recycling provides cleaner fibers, since in this approach a solvent selectively degrades the matrix at lower temperature compared to pyrolysis, with negligible damage to the fibers. Typically, solvolysis in acid media [5] can be performed at moderate temperature (lower than 200 °C) and atmospheric pressure. However, from a process point of view, this approach presents some limitations because concentrated acids are difficult to handle as they pose some safety and environmental issues. The recent introduction of solvents like water or alcohols in critical or supercritical conditions has represented a greener alternative to traditional solvolysis [6,7], but currently costs are still too high to envision an industrial scale up of this technology.

Beside the specific advantages and limitations of the mentioned methods, a common drawback of all these approaches is that the reuse of the matrix is generally not considered. Therefore, these methods are not fully compliant with circular economy principles. More specifically, thermolysis produces a liquid fraction, which is burnt for energy recovery, while liquid residues of solvolysis are complex mixtures of monomers and oligomers that require expensive separation steps for their profitable valorization [3]. This limitation is largely ascribable to the intrinsic nature of the matrix (often an amine-cured epoxy), which is not designed to be soluble or reworkable as a result of its thermosetting network.

Within this framework, the recyclability of crosslinked epoxies has been recently demonstrated by re-designing the polymer network with the introduction of specific functionalities on the monomers, which could form cleavable bonds upon crosslinking. The concept underlies the proprietary technology of Connora [8,9], based on epoxy crosslinked with amine hardeners, which can be cleaved under moderate conditions (70–100 °C) thereby obtaining a thermoplastic epoxy as byproduct. The system was used to recover carbon and flax fibers from epoxy composites [10,11]. Analogous approaches require the introduction of tertiary amine [12] or acetal functionalities [13] into an epoxy cleavable network that could undergo de-crosslinking and allow fiber recovery from acidic media. A step forward in the valorization of the recovered matrix was made possible with the introduction of the concept of covalent adaptable networks (CANs) [14], which enables re-crosslinking the matrix after fiber recovery. In particular, Yu et al. demonstrated full recyclability and repairability of a reversible thermoset composite based on transesterification exchange reactions in the presence of ethylene glycol at 180 °C [15]. Similarly, Wang et al. used ethanol to de-crosslink a reversible novolac boronate network, which enabled recovery of glass or carbon fibers. When ethanol was removed, the matrix could be re-crosslinked [16].

Among the different chemical approaches involving the use of CANs, the Diels–Alder (DA) is a click reaction [17], consisting in the cycloaddition between a 1,3 conjugated diene and a dienophile (i.e., a molecule containing a double or triple bond) [18]. The product of the reaction is a cyclic adduct DA in a dynamic equilibrium with the diene/dienophile. As temperature is increased, the equilibrium can be reverted to the formation of reactants (retro-Diels–Alder reaction) [19]. In particular, the addition reaction between furan (diene) and maleimide (dienophile) is particularly versatile due to its fast kinetics and high yields. Upon proper choice of functional precursors, such diene/dienophile couples can be used to design thermally reversible polymer networks [20,21]. Compared to other CANs, furan/maleimide networks can be prepared from readily available chemicals and exhibit excellent reversibility at mild temperatures in the absence of catalysts, thus making this approach potentially interesting for a wider-scale implementation. DA matrices were demonstrated to enable thermal healability to carbon reinforced composites [22,23,24]. This synthetic tool was also used to impart thermal repairability and recyclability to a wide number of epoxy-based networks [25,26,27,28,29,30,31,32,33,34,35,36,37,38,39,40,41,42,43]. However, the recovery of both fiber and resin component from carbon composites based on the thermal reversibility of epoxy-based DA matrices has not been reported yet.

To bridge this gap, in this work, a DA polymer network was synthetized from the crosslinking of furan-modified epoxides with an aromatic bismaleimide in order to obtain a moldable polymer. After demonstrating the thermal reversibility of such new system, the material was used as a binder for different classes of carbon fibers in order to obtain polymer-based composites. Mechanical properties were assessed by tensile tests and dynamic mechanical analysis (DMA). The fiber components were recovered by solvolysis in common solvents and embedded into new composites. Their quality was assessed by thermogravimetric analysis (TGA) and scanning electron microscopy (SEM). Closing the loop, liquid byproducts of solvolysis were directly employed to produce coatings, which were found to be healable, owing to the intrinsic thermal reversibility of the polymer matrix. To the best of our knowledge, the method proposed in this work represents the first demonstration of the use of DA chemistry to fully recover and recycle fiber and matrix components from a thermoset, without downcycling.

## 2. Materials and Methods

### 2.1. Materials

Bisphenol A diglycidyl ether (DGEBA), N,N-diglycidyl-4-glycidyloxyaniline (DGGO), furfurylamine (FA), 1,1′-(methylenedi-4,1-phenylene)bismaleimide (BM), tetrahydrofuran (THF), propylene carbonate, N,N-dimethylformamide (DMF), and dimethyl sulfoxide (DMSO) were purchased from Sigma-Aldrich (Milan, Italy) while methanol was obtained from Fisher Chemical (Milan, Italy). Reagents and solvents were used as received. Epoxy resin Araldite BY158 and hardener Aradur 2992 CH were provided by Huntsman (Varese, Italy). Milled carbon fibers (PX30, average fiber length 150 μm), carbon nonwoven mats (Optiveil, 20 g/m^2^ area density), and carbon unidirectional fabric (30 g/m^2^ area density) were purchased from ZOLTEK (Nyergesujfalu, Hungary), TFP (Kendal, UK), and R&G Faserverbundwerkstoffe (Waldenbuch, Germany), respectively.

### 2.2. Synthesis of Furan Precursors

Furan derivatives of DGEBA (2F) and DGGO (3F) were synthesized according to a reported procedure [35]. Typically, DGEBA (6.0 g, 17.6 mmol) was dissolved in 10 mL of methanol in a round-bottom flask equipped with magnetic stirrer, reflux condenser, and oil bath for heating. FA (3.43 g, 2:1 molar ratio with respect to DGEBA) was added, and the solution was stirred for 5 h at 85 °C. Upon drying under vacuum, a yellow solid was obtained (bis-furan, 2F). Tri-furan (3F) was obtained as an orange solid by reacting DGGO (6.0 g, 21.6 mmol) with FA (6.3 g, 3:1 molar ratio respect to DGGO) following the same procedure. Yields were nearly quantitative in both cases. Complete conversion of epoxide rings was confirmed by FTIR spectroscopy, monitoring the disappearance of band at 910 cm^−1^ (C–O stretching in the oxirane ring).

### 2.3. Synthesis of Diels–Alder Polymer Networks

**Scheme 1:**
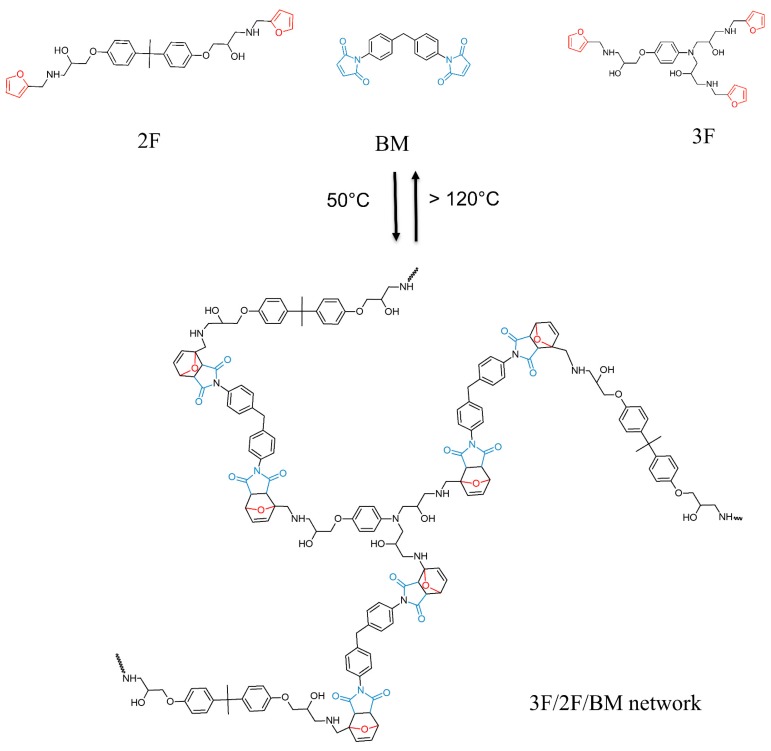
Schematic representation of cross-linking and de-cross-linking of furan-modified bisphenol A diglycidyl ether (DGEBA, 2F) and N,N-diglycidyl-4-glycidyloxyaniline (DGGO, 3F) blends via Diels–Alder (DA) and retro-Diels–Alder (r-DA) reaction with 1,1′-(methylenedi-4,1-phenylene)bis-maleimide (BM).

The 3F and 2F macromers, fed at different molar ratios, were dissolved in THF (20 wt %) in a round-bottom flask equipped with a magnetic stirrer, an oil bath, and a reflux condenser. A stoichiometric amount of BM (considering a 1:1 molar ratio between maleimide and furan groups) was added to the mixture, and the reaction (DA cycloaddition) was allowed to proceed for 24 h under agitation at 50 °C. The obtained gels were dried in a vacuum oven at 50 °C until constant weight and grinded through ball milling. The reaction is depicted in Scheme 1.

### 2.4. Composite Fabrication

Composite films were prepared by compression molding in a JBT Engineering press. Briefly, powdered DA polymer samples were spread in a mold over a polytetrafluoroethylene (PTFE) foil and covered with a sheet of the unwoven mat or the unidirectional fabric. A second layer of polymer powder was spread on the top, and an additional PTFE foil was placed on the sample. The weight ratio between matrix and fibers was 20:1. The stack was heated (1 h at 120 °C or 140 °C), pressed (30 min, 200 bar) and cooled down (2.5 h) to room temperature. Composites with short carbon fibers were prepared by dispersing the fibers in a THF solution of 3F and 2F. Upon addition of stoichiometric amount of BM, the reaction mixture was heated on a hot plate at 50 °C under agitation until most of the solvent was evaporated. The resultant highly viscous liquid was poured in a polydimethylsiloxane (PDMS) mold and dried in a vacuum oven (50 °C, 24 h).

### 2.5. Characterization

DA polymer samples were characterized in terms of infrared spectroscopy, determination of insoluble fraction, and thermal properties. Fourier transform infrared (FTIR) spectra were recorded using a Thermo Nicolet Nexus 670 instrument (Thermo Fisher scientific, Waltham, MA, USA). Measurements were performed on powders dispersed in KBr discs over a spectral range from 4000 to 600 cm^−1^ at a resolution of 4 cm^−1^, averaging 32 scans. For gel content analysis, dried samples (prepared by compression molding) were weighed (w_0_) and immersed in THF (about 70 mL per 0.25 g of material) for 24 h at room temperature under stirring. Samples were removed and dried in a vacuum oven at 50 °C until constant weight (w_f_) was reached. Gel content was then calculated as the percentage ratio between w_f_ and w_0_. Differential scanning calorimetry (DSC) analyses were performed with a DSC 823e Mettler Toledo instrument (Mettler Toledo, Columbus, OH, USA) by applying the following thermal cycle: from 25 °C to 180 °C, from 180 °C to 0 °C, and from 0 °C to 200 °C, with a heating/cooling rate of 20 °C/min. Thermogravimetric analyses (TGA) were performed with a Q500 TGA system (TA Instruments, Milan, Italy) from ambient temperature to 800 °C at a scan rate of 10 °C/min both in air and nitrogen atmosphere. 

Scanning electron microscopy (SEM) images were obtained using a Zeiss Evo 50 EP SEM apparatus (acceleration voltage of 20.0 Kv, Carl Zeiss, Oberkochen, Germany) on freshly crio-fractured surfaces of fibers and composite samples.

Mechanical characterization was performed in terms of tensile tests and dynamic mechanical analysis (DMA). Tensile properties were determined on the DA matrix and on the composites using a Zwick/Roell Z10 universal testing machine (Zwick Roell, Ulm, Germany) equipped with a 10 kN load cell and a long-stroke extensometer. Samples were 50 mm long, 5 mm wide, and 0.3 mm-thick rectangular bars. A strain rate of 0.4 mm/min was applied. For each sample, at least 10 specimens were tested, and the results were averaged. DMA was performed in tension mode, using a Mettler Toledo DMA/SDTA861 apparatus (Mettler Toledo, Columbus, OH, USA). Samples were heated from room temperature to 180 °C at 3 °C/min^−1^ rate and oscillation frequency of 1 Hz.

## 3. Results and Discussion

### 3.1. Synthesis and Characterization of Diels–Alder (DA) Polymer Networks

Furan precursors 2F and 3F were combined in different proportions in order to assess the role of different degree of functionality and reacted with aromatic bismaleimide (BM) in solution (reaction scheme reported in Scheme 1). The formation and precipitation of yellow-to-orange solids from the THF solutions visually gave indication of successful crosslinking. An overview of the different systems prepared is reported in Table 1. Samples have been coded using the targeted average furan functionality (*f_av_*), calculated as follows,

(1) $$f_{av}=\frac{2\cdot n_{2F}\;+\;3\cdot n_{3F}}{n_{2F\;}+\;n_{3F}}$$

where *n*_2*F*_ and *n*_3*F*_ are the number of moles of the bis-furan and tri-furan compounds, respectively.

**Figure 1:**
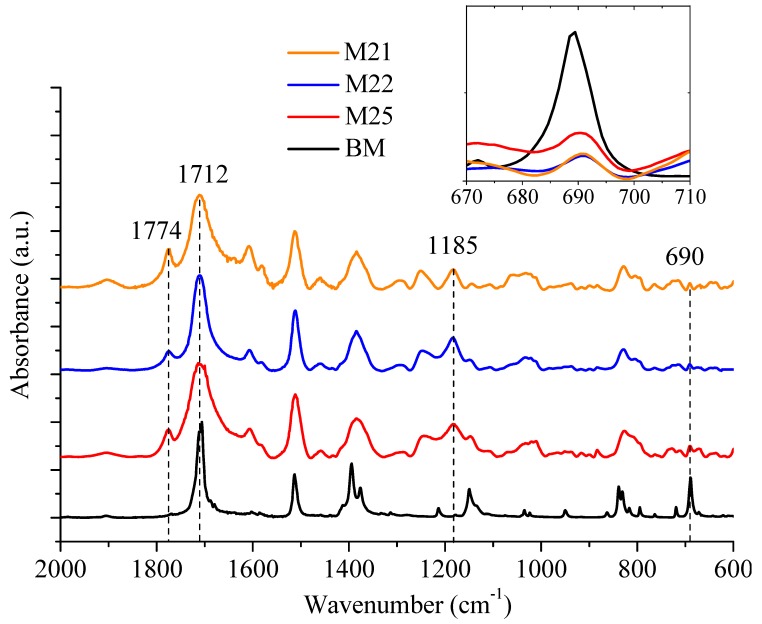
FTIR spectra of crosslinked samples M21, M22, M25 with different furan average functionality, compared to aromatic bismaleimide (BM). In the inset, the peak associated to C–H in maleimide rings (690 cm^−1^) is highlighted.

FTIR spectroscopy was used to assess the occurrence of the DA reaction between the furan precursors and BM. Spectra of the crosslinked samples are shown in Figure 1.

The peaks at 1774 cm^−1^ and 1185 cm^−1^, ascribed to C=O stretching and C–N–C stretching in succinimide rings respectively, are characteristics of DA adducts [28,44,45] and were observed in all the crosslinked polymers, while absent in the BM monomer. Comparing the BM spectrum with the crosslinked polymers, the intensity of the band at 690 cm^−1^ (C–H out-of-plane bending in maleimide rings) strongly decreased (inset of Figure 1), indicating reaction of maleimide groups. 

The extent of DA reaction was estimated in terms of percent ratio of maleimide groups converted, according to the following equation,

(2) $$DA\;conv\;=\;\left(1-\frac{I_{690}^{}/I_{1712}^{}}{I_{690}^{BM}/I_{1712}^{BM}}\right)\times \;100$$

where $I_{690}^{}\;$ and $I_{1712}^{}$ are the intensities of absorption bands of crosslinked samples at 690 cm^−1^ and at 1712 cm^−1^ (C=O stretching of maleimide, used as reference), respectively, while $I_{690}^{BM}\;$ and $I_{1712}^{BM}$ are the intensities of the same bands in the bismaleimide spectrum. Results are reported in Table 1. 

For M25 sample (equimolar ratio of 3F and 2F), a 70% conversion was obtained, significantly higher than the one reported previously on the same material [35], where crosslinking was performed by a thermal treatment at 80 °C in vacuum, up to 10 h. This result could be due to the longer curing steps (24 h in THF and 24 h in vacuum) used in our procedure. In M22 and M21, an increase in the conversion of maleimide groups (up to 85%) was observed. Critical extent of reaction (*p_c_*), that represents the extent of reaction at the gel point was calculated according to the Flory–Stockmayer model [46] from the equation

(3) pc=[1+ρ(f−2)]−12

where f is the number of reactive functional groups on the branch unit (3F in our case), and ρ is the ratio between the number of furan groups in the tri-functional monomer (3F) and the total number of furan groups of the system. Thus, f was a constant and was equal to 3 for all formulations, while ρ varied according to the different stoichiometry. Resulting *p_c_* values indicate that higher conversion at gel point should be obtained by lowering the functionality of the system. Indeed, higher conversions were obtained for M22 and M21 samples, compared to M25. Despite incomplete conversion of maleimide groups, extraction experiments in THF indicated a very high insoluble (gel) fraction confirming the high efficiency of crosslinking achieved through DA addition. M22 formulation can be considered the best compromise between conversion at the gel point and gel fraction and was selected for most of the following experiments.

### 3.2. Thermal Reversibility

**Figure 2:**
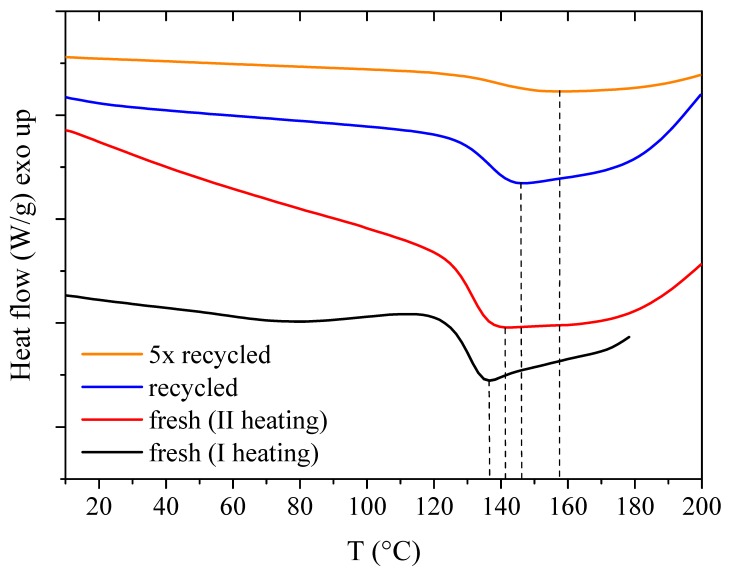
Differential scanning calorimetry (DSC) traces (heating scans) of M22 DA polymer network: Freshly synthetized (2 consecutive thermal cycles); compression molded; after five molding processes. Dashed lines indicate peak temperatures of retro-Diels–Alder.

DSC measurements were performed in order to study the thermal behavior of the DA polymer as a function of multiple thermal cycles. Heating scans on M22 samples with different thermal histories are reported in Figure 2. 

The curve recorded during the first heating scan on the freshly synthetized material showed a broad endothermic transition in the 120–180 °C temperature range, which is related to the retro-Diels–Alder (r-DA) reaction. After cooling and re-heating the sample in the DSC apparatus (II scan), the transition (while slightly shifted to higher temperature) was still present, thus confirming the reversibility of the crosslinking process [35]. 

Thermal reversibility was practically established by processing the crosslinked polymer by compression molding, where a longer thermal cycle (1 h at 120 °C, to allow de-crosslinking via r-DA, followed by slow cooling to room temperature for DA) was applied, compared to DSC scans. Traces of molded materials showed that the polymer network was still able to undergo r-DA transition. Moreover, pressed samples were repeatedly recycled by grinding and re-molding under the same conditions, up to 5 times. DSC traces still exhibited a r-DA transition, proving the ability of the system to retain its reversibility. The minimum observed in the r-DA temperature interval (T_rDA_) shifted to higher temperature values over consecutive thermal cycles, suggesting an increasing chemical fatigue in the DA-rDA process. This trend has been reported for similar furan/maleimide networks [47] and ascribed to a change in the ratio between the two possible conformers of DA adducts [48] or to the occurrence of other side reactions [37,49]. 

Further proof of thermal reversibility was obtained from simple gelation tests. The 2F and 3F precursors are soluble in DMF at room temperature. On the other hand, once crosslinked with BM, the resulting DA polymer becomes insoluble under the same conditions. Upon heating at 120 °C, complete dissolution was achieved in 10 min (de-crosslinking through r-DA). The solution was cooled down to room temperature and underwent gelation overnight (crosslinking through DA). Analogous reversibility was observed in DMSO and propylene carbonate solutions.

### 3.3. Composites Preparation and Characterization

**Figure 3:**
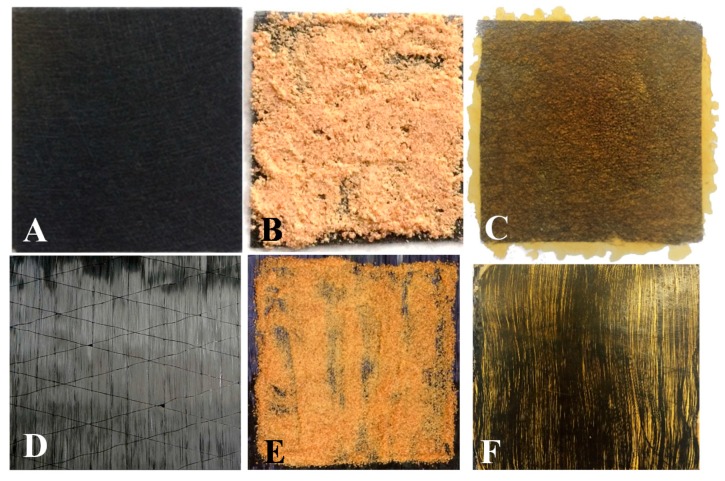
Steps of composites preparation: (**A**) Nonwoven mat, (**D**) unidirectional fibers covered with a layer of powdered DA polymer (**B**,**E**) and after compression molding (**C**,**F**).

Composite films were prepared by compression molding (30 min, 120 °C, 200 bar followed by slow cooling) a stack made of a foil of fibers (nonwoven mat or unidirectional) between two layers of finely grinded MA22 (DA polymer with *f_av_* = 2.2), as shown in Figure 3. A matrix/fiber weight ratio of 20:1 was applied in order to visually impregnate and completely cover the fiber foils surfaces. Upon hot pressing, the matrix forms a smooth and transparent layer, and it is possible to see the reinforcement texture beneath. This can be an indication of satisfactory impregnation due to the lowering in viscosity caused by the r-DA de-crosslinking [22].

**Figure 4:**
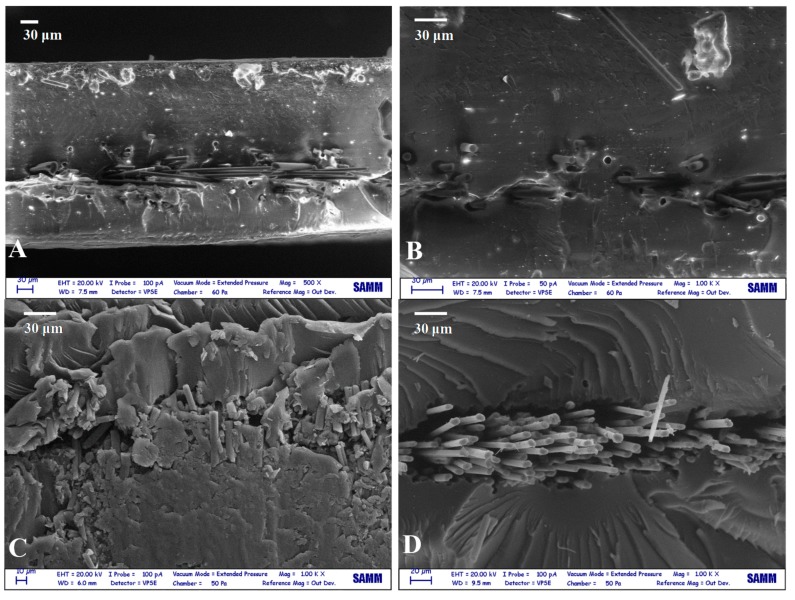
Cross-sectional SEM micrographs of fractured surfaces of DA polymer composites with (**A**,**B**) nonwoven carbon mat and (**C**,**D**) unidirectional carbon fibers.

A study on the morphology of the two types of composites, reinforced with nonwoven mat or with unidirectional fibers, was carried out by means of scanning electron microscopy (SEM) on freshly fractured surfaces (Figure 4). 

As can be observed in Figure 4A, the DA/nonwoven mat composite has a sandwich-like structure, where a layer of fibers is embedded between two layers of DA resin. At higher magnification (Figure 4B) it can be noticed that the surface of fibers looks neat, and there are some voids in the matrix phase, possibly resulting from pulling out of fibers during fracture. These features suggest that, while impregnation is good enough, the fiber/matrix adhesion is not optimal. As for the DA/unidirectional fibers composite, impregnation was not homogeneous along the fractured surface. In some points fibers, are dispersed in the matrix phase (Figure 4C) while in other regions the matrix does not wet the bundle of fibers (Figure 4D). 

**Figure 5:**
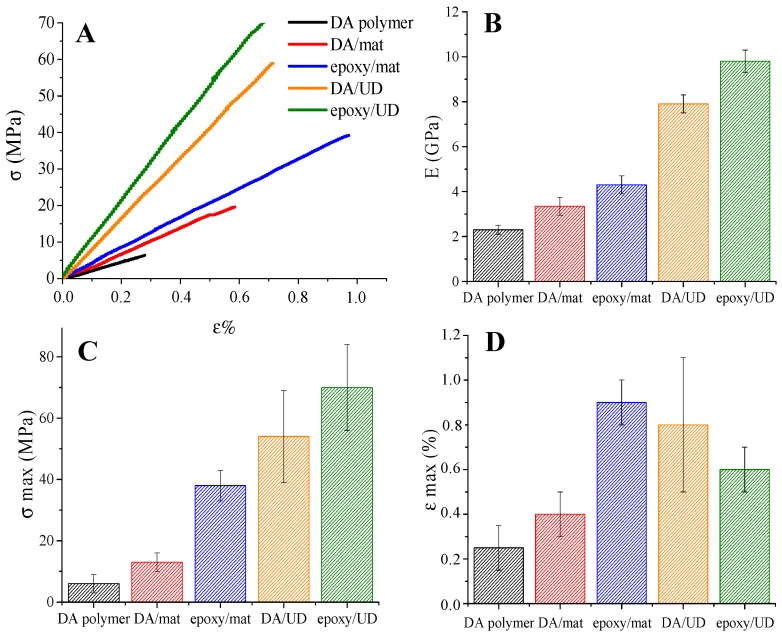
Tensile test results for neat DA polymer and its composites with nonwoven carbon mats (DA/mat) and unidirectional carbon fibers (DA/UD) compared to reference composites (epoxy/mat and epoxy/UD): (**A**) Median stress-strain curves, (**B**) Young’s modulus E, (**C**) tensile strength σ_max_, and (**D**) maximum strain ε_max_.

Mechanical properties of the composites were determined through tensile tests and were compared to reference composites prepared from commercial epoxies (Figure 5). Notably, oligomeric DGEBA (Araldite BY158) and amine crosslinker (Aradur 2992 CH) were mixed (100:34 *w*/*w*), roll painted on both sides of fiber foils (nonwoven mat or unidirectional) and cured for 2 h at 100 °C.

Representative stress-strain curves (Figure 5A) show that all samples broke without apparent plastic deformation. In particular, the neat DA polymer has a tensile strength (σ_max_) of 6 MPa, similar to the one reported in the previous study [35]. Reinforcement provided superior properties, as expected, markedly with unidirectional carbon fibers (DA/UD). Given that stress was applied along the orientation of the fibers, unidirectional fabric provided better reinforcement than randomly oriented ones in the mat. To rationalize from a theoretical standpoint the effect of the reinforcing fibers on the mechanical properties of DA/UD composites, a theoretical value for Young’s modulus was calculated (*E_c_*), according to a rule of mixtures model [50],

(4) $$E_{c}=\varphi \cdot E_{f}+\left(1-\varphi \right)\cdot E_{m}$$

where *φ* is the volume fraction of the fibers and *E_f_* and *E_m_* are the moduli of the fibers and of the matrix, respectively. For DA/UD samples, *φ* was found to be 0.03 (calculated from the densities of the fibers, 1.85 g/cm^3^ and of DA polymer, 1.15 g/cm^3^), *E_f_* was 230 GPa (according to the specifications provided by the manufacturer), and *E_m_* was 2.3 GPa (as determined experimentally). Therefore, a maximum theoretical modulus of 9.16 GPa was calculated, in close agreement with the measured value. Furthermore, mechanical properties obtained from the reference samples (epoxy/mat and epoxy/UD) are similar but systematically higher than the DA composites, possibly due to the higher content of embedded fibers (6.7 wt %).

**Figure 6:**
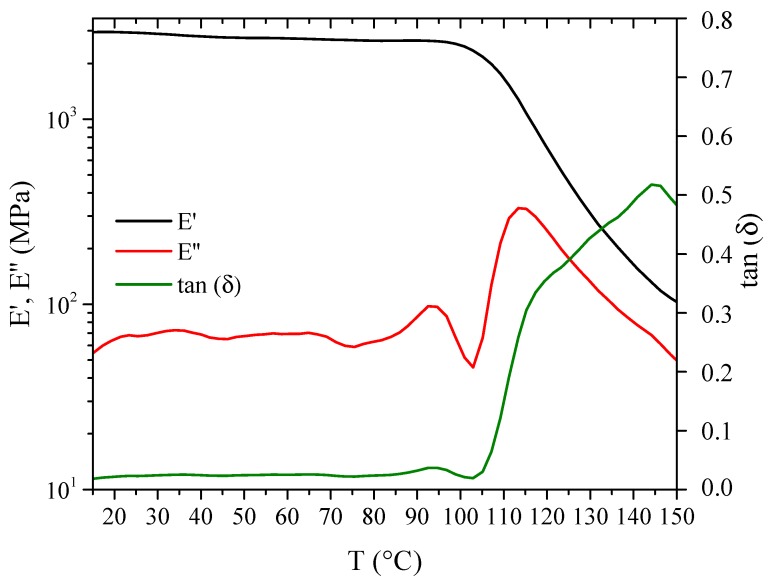
Storage modulus E’, loss modulus E’’ and damping factor tan δ from Dynamic mechanical thermal analysis (DMTA) of M22 DA polymer reinforced with nonwoven mat.

Dynamic-mechanical thermal analysis (Figure 6) was carried out on the composites in order to assess the response of storage modulus (E’) and damping factor (tan δ) to a temperature increase (20–180 °C) during oscillatory loading.

The plot of the storage modulus exhibits a well-defined plateau up to 100 °C, followed by a drop due to a transition from elastic to viscous behavior, ascribed to the r-DA reaction. Indeed, the softening temperature, determined as the peak of the dissipative factor tan (δ) of the matrix component (T = 145 °C), nicely corresponds to the T_rDA_ peak observed in DSC traces (Figure 2). The transition confirms thermal reversibility of crosslinked matrix while establishing an upper limit in the application temperature of the composite. This limitation is characteristic of reversible thermosets based on furan/maleimide DA chemistry [21].

### 3.4. Fiber Recovery through Solvolysis and Reuse

Recovery of the carbon fibers was performed by a solvolysis-like approach, based on the evidence of reversibility of crosslinks in the DA matrix, gained from reversible gelation experiments. Three different classes of carbon reinforcements were recovered: Milled fibers from the solution-cast composites, non-woven mat, and unidirectional fabric from compression-molded composites.

**Figure 7:**
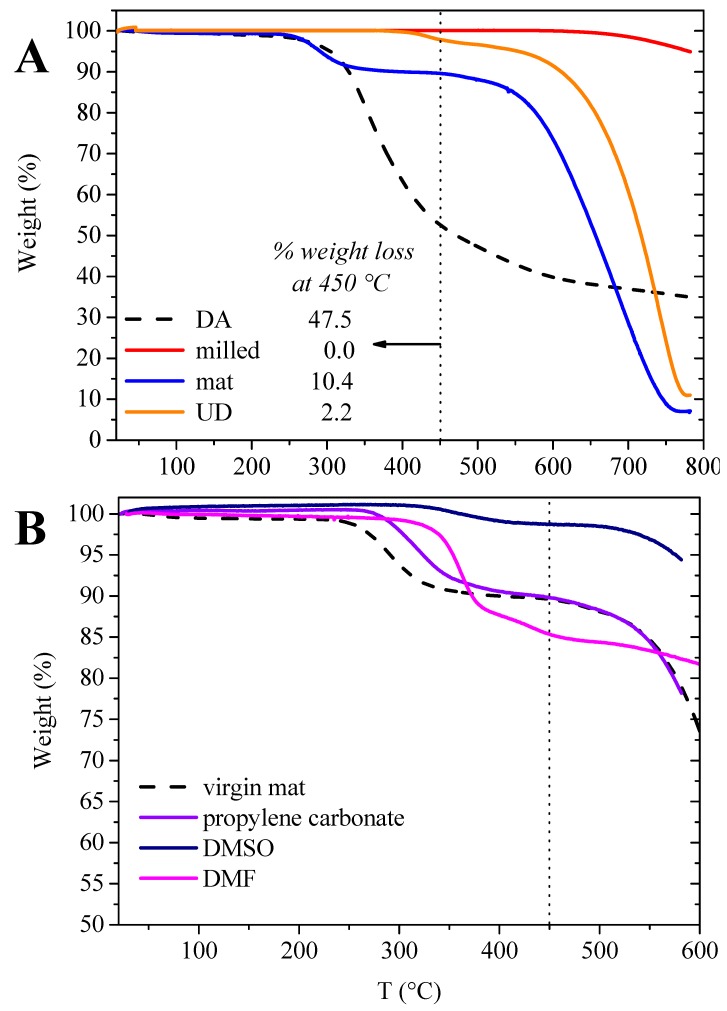
(**A**) TGA plot of neat matrix (DA polymer) compared to the neat carbon fibers: Milled, nonwoven mat, and unidirectional. Insert: Mass loss values recorded at 450 °C; (**B**) expanded TGA plot of nonwoven mats recovered through solvolysis in different solvents compared to virgin. TGA performed in inert atmosphere.

Solvolysis experiments were performed by immersing samples of the different composites in different high boiling solvents (about 10 ml per gram of sample), such as DMF, DMSO, and propylene carbonate. Upon raising the temperature up to 120–125 °C (r-DA range) the solvent turned to orange-to-brown color. After 30 min, black solids (mostly fibers) were collected by hot filtration over a PTFE filter and dried in a vacuum oven at 50 °C until constant weight was reached. A very high recovery yield (>96%) was achieved in all the tests. Purity of recovered fibers was estimated by TGA measurements in inert atmosphere, preliminarily performed on the neat components (Figure 7A). 

The thermogram of the matrix exhibits a single major decomposition event in the 350–450 °C range, assigned to the parent epoxide monomer [51]. Residual weight (34.9%) recorded at 800 °C indicates the formation of char due to the non-oxidative environment. This behavior has also been reported for commercial epoxy resins [52] as well as for epoxy-based polymer networks based on DA chemistry [36]. As for the reinforcements, milled fibers significantly degrade above 600 °C, owing to the very high C content (>99%) as declared by the manufacturer, while nonwoven mats and UD fibers start to decompose at lower temperatures due to the presence of organic binder or sizing. As a result, the volatile content at a selected reference temperature (450 °C) significantly changes with the type of fibers (insert of Figure 7A). Those values were compared with the weight loss of recovered fibers at the same temperature, which are reported in Table 2 along with the experimental conditions used for the solvolysis tests.

Milled fibers exhibit a very low volatile content (2.1%) after recovery, clearly ascribed to the presence of matrix residues. TGA curves of recovered mats from all the solvents are comparable with the curve of the virgin mat (Figure 7B). In particular, for the mat recovered from DMSO, the volatile content at 450 °C is even lower than the virgin mat, suggesting that DMSO was able to remove part of the organic binder. However, the degradation step of the fiber sizing agent is overlapped with the one of the matrix, thus it is not possible to ascribe this weight loss to the sizing or to the resin residue uniquely. Since degradation of the matrix at 450 °C is not complete (Figure 7A), weight loss values would represent just a fraction (47.5%) of the total residue on the fiber. Therefore, more conservative estimates of the matrix residue on the fibers were calculated (Table 2), taking into account these considerations.

Overall, the purity of reclaimed fibers appears comparable to that obtained by means of low or high temperature solvolysis procedures [53,54] and the more recent processes based on critical or supercritical fluids [55,56,57,58]. Indeed, the recovery efficiency is high, considering that fibers were recovered in a single step, batch process, while some of the most efficient solvolysis processes entail semi-continuous conditions [58] and/or several washing steps [57] in order to reach low matrix residues (<5 wt %). 

**Figure 8:**
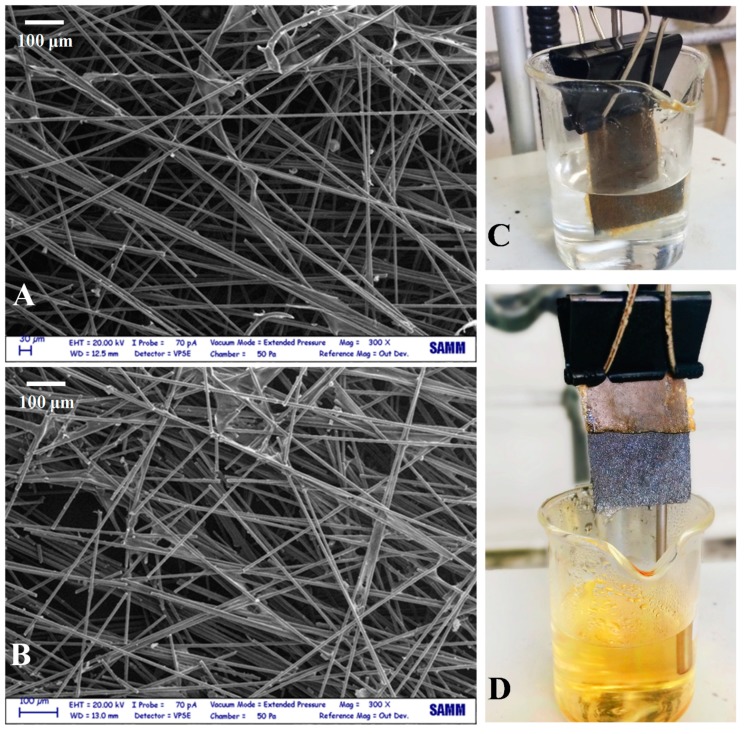
Solvolysis proof-of-concept: Composite sample partially immersed in solvent at room temperature (**A**) and removed after washing at 120 °C (**B**). SEM images of virgin nonwoven mat (**C**) compared to the one recovered from solvolysis in propylene carbonate (**D**).

A visual evidence of the fiber quality was obtained by SEM analysis. Specifically, images recorded on the virgin non-woven mat (Figure 8A) confirmed the presence of the organic sizing wetting the randomly oriented fibers. As a representative example, recovered samples from propylene carbonate are found to contain matrix residues (Figure 8B), in agreement with the TGA results previously discussed.

The simple setup shown in Figure 8C allowed to recover the nonwoven mat reinforcement as a whole, with no visible damage. After drying (50 °C, 24h) the mat was directly (re-)used in order to fabricate a composite by hot pressing between two layers of freshly powdered DA polymer, as described above. The obtained material was not visibly damaged by reprocessing. Tensile tests exhibited a Young’s modulus of 3.2 GPa (97% of the original value) and a maximum stress of 8 MPa (13 MPa in the virgin composite). The reduction in the tensile strength suggests the need for reprocessing the fibers prior to further use in second generation composite by adding a suitable sizing that could possibly improve adhesion with the matrix [3]. An additional recovery in propylene carbonate was performed on the composite with the reprocessed mat. A 10.4% weight loss (10.2% after first solvolysis) detected at 450 °C by TGA analysis in inert atmosphere substantially confirmed the reproducibility of the recovery approach based on the DA chemistry proposed here.

To assess the influence of the chemical composition of the matrix on the efficiency of the fiber recovery process, solvolysis tests were also performed on composites based on M25 as matrix (DA polymer with *f_av_* = 2.5) and non-woven fibers as reinforcements. For the fabrication of these composites, a compression-molding temperature as high as 140 °C (20 °C higher than the one used to fabricate M22 composites, but still in the r-DA temperature range) was necessary in order to obtain homogeneously and completely covered mats. The higher functionality of M25 required more severe processing conditions compared to M22, in order to ensure proper flow of the material. The recovery process performed in DMSO and DMF at 120–130 °C resulted in visually impure carbon mats covered with insoluble gelled material. TGA measurements detected a weight loss of 30.0% and 55.4% at 450 °C after solvolysis in DMF and DMSO respectively, confirming the high content of the residual matrix on the fibers. This behavior can be attributed to the occurrence of side reactions during the composite fabrication that could lead to formation of irreversible crosslinks. Indeed, given the temperature employed and the presence of amine groups in the macromers and maleimide moieties from BM at the applied temperature, Michael addition and/or radical polymerization cannot be excluded [22].

### 3.5. Matrix Reuse as a Smart Coating

**Figure 9:**
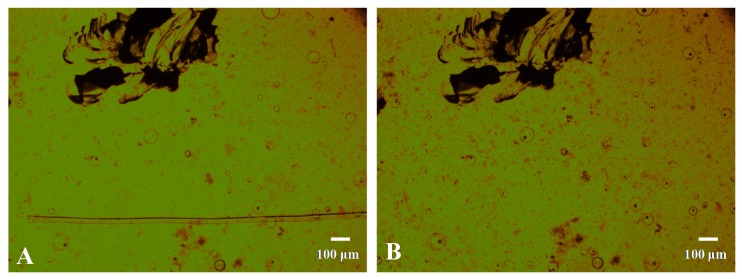
Optical microscope images of coating obtained from recovered DA polymer: (**A**) Damaged by scalpel scratching and (**B**) after thermal treatment at 120 °C and slow cooling to room temperature.

Solvolysis of composites resulted in the production of DA polymer solutions (with an average concentration of about 0.1 g/mL) which, upon cooling to room temperature, spontaneously turned to swollen gel (DA crosslinking). A potential reuse of such recovered DA polymer solutions as smart repairable coatings was studied in this work by using a simple procedure that exploits thermal reversibility coupled with the presence of the solvent used during the recovery. Thus, gels of DA polymer swollen in DMF were heated to 120 °C until complete solubilization was achieved (r-DA de-crosslinking). The so-obtained hot solutions of DA polymer in DMF were drop cast on pre-heated glass slides. After the evaporation of most of the solvent, the obtained coatings were treated at 50 °C in vacuum until constant weight was reached in order to ensure complete drying and crosslinking through DA. In order to check their thermal repairability, coatings were manually scratched with a scalpel (Figure 9A) and healed upon thermal heating followed by slow cooling to room temperature. The scratch completely disappeared after heating at 120 °C for 1 h (Figure 9B), due to the ability of the polymer to flow when the de-crosslinking (r-DA) temperature range is reached. 

Interestingly, the matrix material retains its reversibility after several thermal cycles (namely compression molding, recovery in solvent, redissolution). This represents a clear advantage compared to common thermochemical recycling processes, where the resin is irreversibly degraded during the recovery of the fibers [6]. Furthermore, differently from cleavable epoxies [8,11,12,13] the matrix is recovered, while preserving the ability of being re-crosslinked and directly re-employed, without the need of being separated from the solvent, reprocessed, or added with fresh material or curing agents. Finally, compared to other CANs approaches, re-crosslinking does not need expensive or unstable catalysts [59] and results in a coating with the added value of self-healing, which, paired with its epoxy-based nature, makes it attractive for potential corrosion protection application [60].

## 4. Conclusions

An innovative approach towards an efficient circular economy of carbon reinforced thermoset composites is presented. A polymer network, synthetized from the reaction of furan-modified commercial epoxides with a bismaleimide through DA addition, was employed as the matrix phase. 

Thermal reversibility of the polymer network, demonstrated by solubility tests and DSC, allowed to embed carbon nonwovens or fabrics in the matrix. Most notably, reversible crosslinking was the key to reclaim the fiber content and the matrix at the same time through a one-step, selective dissolution in common solvents. On one hand, purity of recovered fibers, assessed by TGA and SEM, reached 95% and allowed reprocessing into composites with retention of Young’s modulus compared with the fresh ones. On the other hand, a first demonstration of direct reuse of the solution containing the residual matrix in the production of a thermally healable coating was given.

In conclusion, the proposed recycling strategy, based on the intrinsic DA reversibility of the matrix, enables higher sustainability than traditional solvolysis methods, since it employs common solvents, in milder conditions of temperature, with full recovery of the composite components.

## Figures and Tables

**Table 1:** Average furan functionality of 2F/3F blends and properties of the DA networks obtained after crosslinking with BM, namely predicted critical extent of reaction (*p_c_*), Diels–Alder conversion, and insoluble fraction in THF.

Sample	2F:3F (mol:mol)	*f_av_*	*f*	*ρ*	*p_c_* ^a^	DA Conv ^b^ (%)	Gel Content (%)
M25	1:1	2.5	3	0.60	0.79	70 ± 1	98.5 ± 0.5
M22	4:1	2.2	3	0.27	0.89	85 ± 2	98.6 ± 0.8
M21	9:1	2.1	3	0.14	0.94	85 ± 2	96.5 ± 0.5

^a^ predicted according to Flory–Stockmayer model ^b^ calculated from FTIR.

**Table 2:** Solvolysis tests on M22 composites and results of TGA on the recovered fibers, namely weight loss at 450 °C and estimated amounts of residual matrix. TGA performed in inert atmosphere.

Fiber Type	Solvent	Weight Loss at 450 °C (%)	Estimated Matrix Residue (%)
milled	DMF	2.1	4.4
mat	DMF	14.7	31.0
mat	propylene carbonate	10.2	21.5
mat	DMSO	2.3	4.9
UD	propylene carbonate	17.7	37.4
